# Laparoscopic surgery combined with the double-J tube implantation for the rare cystic-solid schwannoma of seminal vesicle: A Case Report and Literature Review

**DOI:** 10.1097/MD.0000000000029352

**Published:** 2022-07-15

**Authors:** Canbin Lin, Aidi Liang, Shulin Liang, Xiao Wang, Lei Meng, Ming Chen

**Affiliations:** a Department of Urology, The First Affiliated Hospital of Guangzhou University of Chinese Medicine, Guangzhou, Guangdong, P.R. China; b The First Clinical Medical College, Guangzhou University of Chinese Medicine, Guangzhou, Guangdong, P.R. China; c Department of Traditional Chinese Medicine, Jinan University, Guangzhou, Guangdong, P.R. China.

**Keywords:** double-J tube, rare, laparoscopic surgery, schwannoma, seminal Vesicle

## Abstract

**Rationale:**

Schwannoma is common in young and middle-aged people and occurs in the head, neck, posterior mediastinum, and retroperitoneal. Schwannoma, on the other hand, is a rare occurrence in the seminal vesicle. Early diagnosis and treatment are crucial since the disease can cause lower abdominal pain, nocturia, frequent urination, blood sperm, and other symptoms. There is no standard diagnostic or treatment guideline for seminal vesicle schwannomas currently. Therefore, the treatment experience relies on the few cases reported throughout the world.

**Patient concerns:**

A 45-year-old male patient discovered that the tumor beside the right side spermatophore is bigger than 3 years ago.

**Diagnosis:**

Schwannoma of seminal vesicle.

**Interventions:**

Ureter double-J tube implantation and laparoscopic surgery for schwannoma of seminal vesicle.

**Outcomes:**

The operation process went smoothly. And the patient was no discomfort after half a year.

**Conclusion:**

Schwannoma of the seminal vesicle is very rare in the clinic, and the imaging examination was not conclusive. The diagnosis mainly depends on pathological results. Surgical resection is the best treatment method for schwannoma. In surgery for schwannoma of seminal vesicle, combined with the ureter double-J tube implantation are many benefits. This case is an excellent example of the seminal vesicle schwannomas.

## 1. Introduction

Schwannoma is a tumor of the peripheral nerve sheath made up of Schwann cells.^[1]^ It is usually found in the head, neck, posterior mediastinum, and retroperitoneal.^[2]^ The etiology of schwannoma is not very clear, which occurred in unilateral, benign, and slow growth were common.^[3]^ Malignant schwannoma is related to the multiple neurofibromas, and deep schwannoma is more likely to be malignant than the superficial type.^[4]^ Schwannoma is mostly asymptomatic, but when it is big enough, it can produce corresponding symptoms by compressing the adjacent organs.^[5]^ B-ultrasound, CT, and MR are helpful to the diagnosis of the tumor: B-ultrasound often shows well-defined and homogeneous hypoechoic nodular mass. CT showed clear boundary and high-density solid mass.^[6]^ To confirm the diagnosis, a biopsy is performed.^[7]^

However, schwannoma in the seminal vesicle is rare in the clinic, which has been reported since 2002.^[2,8]^ There are just 14 reports on the diagnosis and treatment of seminal vesicle tumors.^[2,3,5,7,9–18]^ When the schwannoma of the seminal vesicle is small, most patients are asymptomatic and often found by physical examination. When the size of the tumor is large enough to compress the surrounding structures, it causes hydronephrosis, hematospermia, and infertility.^[5]^

Nowadays, there is no guideline of schwannoma in seminal vesicles in the world. Most of the existing reports of treatment are open surgery or laparoscopic surgery.^[7,16]^ When the schwannoma of the seminal vesicle is large or close to the ureter, the procedure becomes difficult and the ureter is easily damaged. It will result in complications such as ureteral fistula. Use a cystoscope to keep a double-J tube in the ureter before opening or laparoscopic surgery to protect the ureter and show the ureter clear during surgery.^[19]^ Thus, it can reduce the difficulty of the operation and postoperative complications.

## 2. Case Report

**Figure 1.**
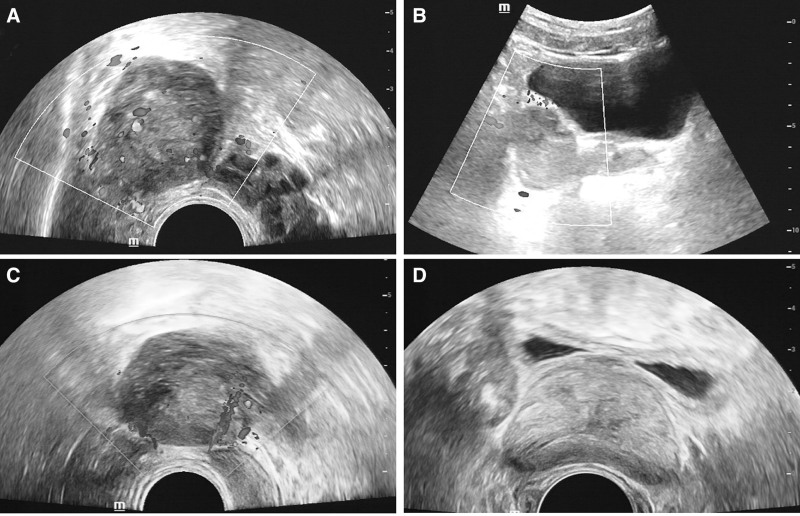
(A) Color Doppler ultrasound indicated a solid mass with rich blood flow beside the seminal vesicle on the right side of the pelvis. (B) The right side wall of the bladder and the right seminal vesicle gland are slightly pushed. (C) Color Doppler image after 2 months. (D) Color Doppler image after 2 months.

**Figure 2.**
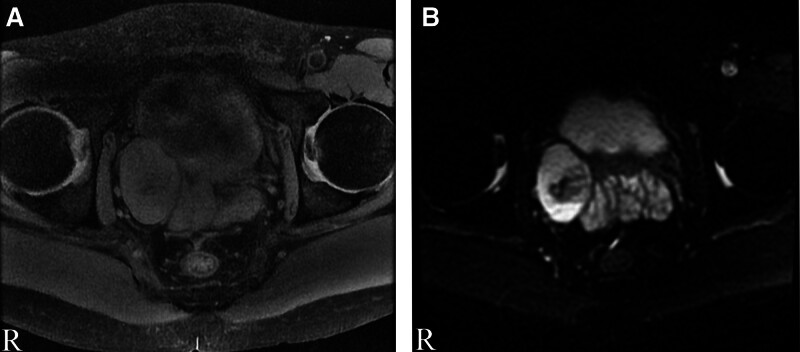
(A) MRI (T1WI) scan indicated an oval-shaped solid mass next to the seminal vesicle gland, about 5.3 cm × 3.4 cm in size. (B) DWI indicated high signal intensity of the mass. DWI = diffusion-weighted imaging, T1WI = T1 weighted image.

**Figure 3.**
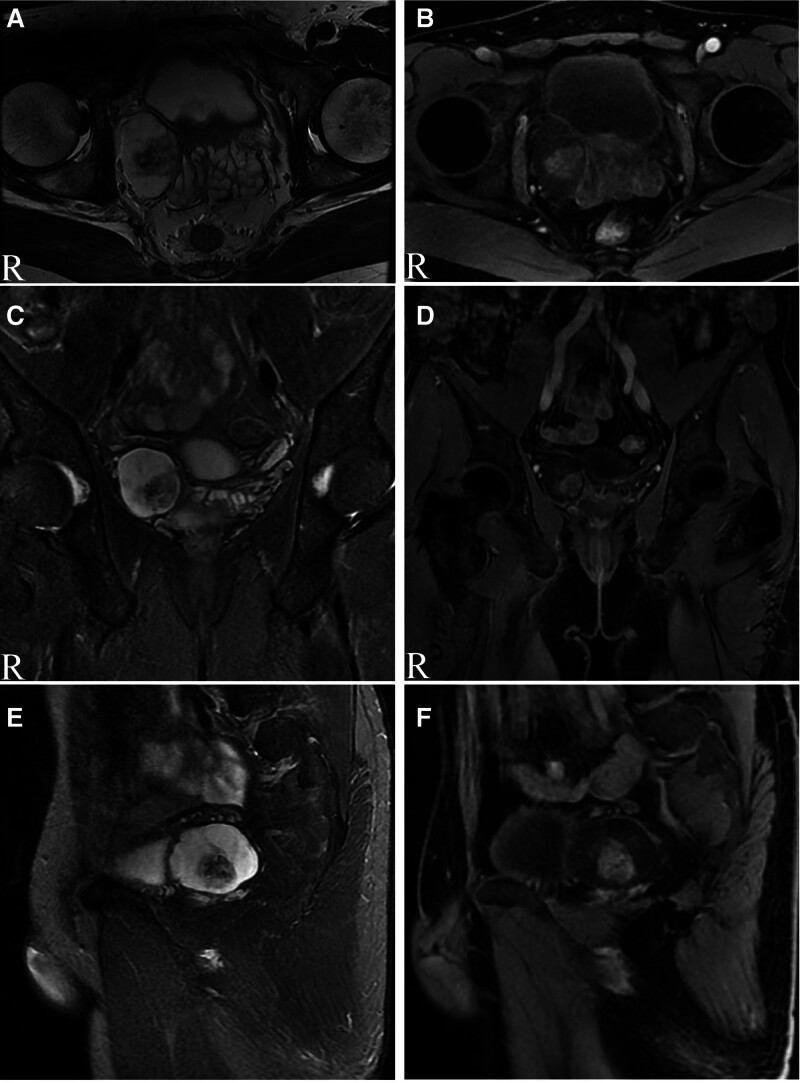
(A) Cross section (MRI T2WI) suggests that the mass is slightly higher and low confounded signal, and the prostate, the right seminal vesicle gland and the right wall of the bladder are compressed. (B) Cross section with enhancement showed that the tumor was slightly uneven and continued to strengthen and the cyst wall was slightly strengthened. (C) Coronal plane (MRI T2WI). (D) Coronal plane with enhancement. (E) Sagittal plane (MRI T2WI). (F) Sagittal plane with enhancement.

A 45-year-old man found a tumor beside the right side spermatophore in the 2017 July physical examination, and he did not pay attention to it because he was without any symptoms. About 3 years later, the patient returned to the hospital for a checkup, and the color Doppler Ultrasound was performed in August 2018. The color Doppler Ultrasound indicated a solid mass with rich blood flow beside the seminal vesicle on the right side of the pelvis, about 43 mm × 31 mm in size (Fig. 1A). The right side wall of the bladder and the right seminal vesicle gland was slightly pushed (Fig. 1B). After 2 more months of observation, the patient was taken to the hospital, since an ultrasonic examination indicated that the mass was slightly larger than previously, measuring 49 mm × 38 mm (Fig. 1C,D).There was no obvious abnormality found in the results of blood, urine, and feces examination. To evaluate the information of the mass, magnetic resonance imaging was performed. MRI scans showed a type of elliptical cystic-solid mass shadow on the right side of the seminal vesicle gland, with a size of about 5.3 cm × 3.4 cm (Fig. 2A). Diffusion-weighted imaging indicated the high signal intensity of the mass (Fig. 2B). MRI T2 weighted imaging suggested that the mass is a slightly high and low confounded signal, and the prostate, the right seminal vesicle gland, and the right wall of the bladder are compressed (Fig. 3A). The enhancement scan showed that the tumor was slightly uneven and continued to strengthen and the cyst wall was slightly strengthened (Fig. 3B). The coronal T2 scan and enhanced scan are shown in Figure 3C,D, respectively. Moreover, the sagittal T2 scan and enhanced scan are shown in Figure 3E,F, respectively.

**Figure 4.**
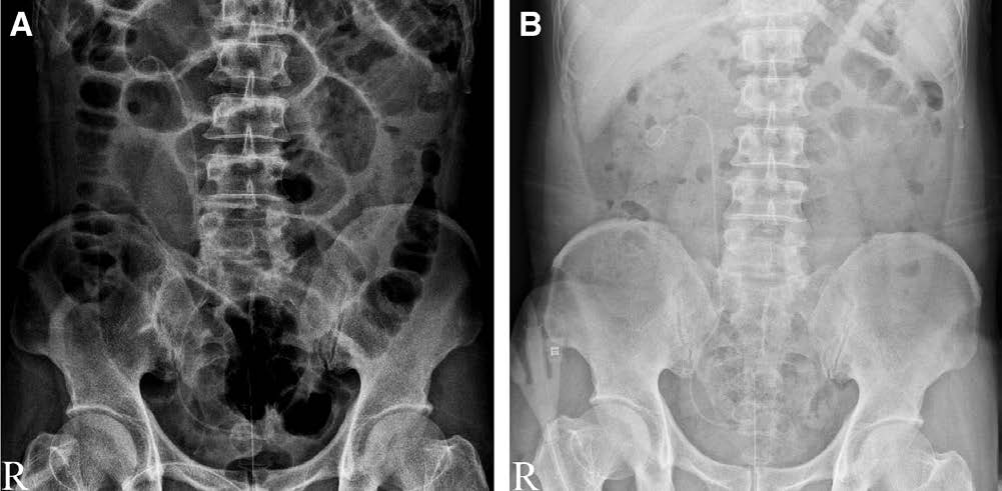
(A) KUB scan (October 28, 2018) indicated that the double-J tube is in good position after the operation. (B) KUB scan (December 8, 2018) indicated that the double-J tube is in good position after the operation. KUB = kidney ureter bladder.

**Figure 5.**
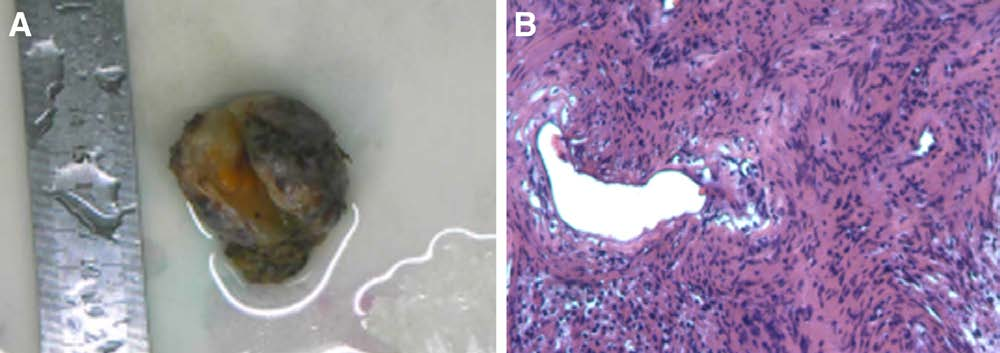
Pathological examination demonstrated that the mass is composed of a loose cell area and a dense area. Among them, the cell morphology is mild, and some are arranged in a fence shape (hematoxylin and eosin staining; magnification, ×200). Immunohistochemistry showed: S-100(+), Ki-67(+<1%).

According to the above-mentioned findings, the patient’s ureter was very close to the seminal vesicle tumor, and because the volume is so large, the ureter is easily damaged during surgery. Our main emphasis has changed to how to protect the ureter. According to the experience of complex pelvic surgery, double-J tube placement before the operation is helpful to protect the ureter. Thus, the patient underwent right ureter double-J tube implantation and laparoscopic right seminal vesicle tumor resection in general anesthesia. The operation process went smoothly, and the operating time about 275 minutes. The amount of intraoperative blood loss was small, about 5 mL. Postoperative review of kidney ureter bladder scan (Fig. 4A) showed the positive position of the double-J tube and good continuity of the ureter. The tumor specimen is shown in Figure 5A. Pathological examination (Fig. 5B) showed that the mass is composed of a loose cell area and a dense area. Among them, the cell morphology is mild, and some are arranged in a fence shape (hematoxylin and eosin staining; magnification, ×200). Immunohistochemistry results S-100(+), Ki-67(+<1%).

The patient recovered well with no any complication, and the hospitalization time was 13 days. More than a month later, a new double-J tube replacement was performed to continue dilating the ureter (Fig. 4B depicts the replaced double-J tube). After another month of ureteral dilation, the double-J tube was removed. Finally, the patient had no discomfort in 3 months follow-up period. The patient has provided informed consent to open cases.

## 3. Discussion

Schwannoma of the seminal vesicle is very rare in the clinic, and there are just 14 case reports^[2,3,5,7,9–18]^ could be searched in full text from PubMed database (https://pubmed.ncbi.nlm.nih.gov/). The clinical characteristics of 14 cases were shown in Table 1. Most schwannomas are benign and malignant schwannomas are extremely rare, but malignant schwannomas have been recorded.^[4,20,21]^ In general, for the diagnosis of the seminal vesicle schwannoma, CT examination often showed a well-defined high-density solid mass.^[1,6,14]^ But the pelvic MR showed a round cystic-solid mass in this case, indicating that the imaging examination was inconsistent. Therefore, identifying the nature using imaging is challenging, and Pathology is the gold standard diagnosis of schwannoma.^[7,22,23]^ Pathological and immunohistochemical analysis can show the origin of schwannoma. Schwannomas are mainly composed of Antoni A and Antoni B regions.^[22]^ The cells in the area of Antoni A were closely arranged and showed a whirlpool or onion skin-like structure.^[22,24]^ The cells in the Antoni B area were arranged loosely and disorderly, with many vacuoles or watery liquid, forming microcapsules or large cavities (19). The degree of cytological atypia was not obvious.^[22,24]^ The expression of S-100 protein was diffuse and the Ki-67 labeling index was <1%.^[7]^

**Table 1:** The clinical characteristics of 14 cases from the PubMed database.

**Characteristics**	**Total**	**No. of patients(%**)
**Age(Years**)		
≤60	11	79.57%
>60	3	21.43%
**Tumor size(cm**)		
≤4.0	6	42.86%
>4.0	8	57.14%
**Nature**		
Solid	10	71.42%
Cystic	2	14.29%
Cystic solid	2	14.29%
**Operation mode**		
laparoscopic	6	42.86%
open surgery	7	50.00%
conservative	1	7.14%

The treatment strategy of seminal vesicular schwannoma mainly depends on attitude, tumor condition, overall health status, and symptoms of the patients. At present, according to the relevant literatures reported,^[3,5,7,10,14,18]^ the outcome of conservative treatment for seminal vesicular tumors remains unknown, and majority of experts agree that surgical resection is the first and the best choice of treatment. In surgical treatment, there are many reports of traditional open surgery.^[2,9,11,13,14,17]^ The seminal vesicle is located deep in the pelvic cavity, so open surgery has the disadvantages of poor exposure, large trauma, and long time, and is easy to be complicated with side injuries such as rectum, bladder, and ureter. Compared with traditional open surgery, laparoscopic surgery has some advantages in the removal of seminal vesicle schwannomas. In laparoscopic surgery,^[3,5,7,10,15,16]^ the field of vision of the tumor and its surrounding anatomical structure is enlarged and it is safer to peel off tumors and surrounding tissues. In addition, the anatomical position of the seminal vesicle is located in the deep pelvic cavity, adjacent to the ureter. When the tumor is near the seminal vesicle, the operation runs the risk of damaging the ureter. But if the tumor cannot be completely removed, it will increase the risk of implantation and recurrence. It has been reported that prophylactic placement of double-J tube is widely used in pelvic floor surgery (Urology, Anorectum, and Gynecology), which can better expose the ureter and reduce the risk of ureteral injury.^[19,25,26]^ Thus, it is recommended that double-J tubes be placed prophylactically before the operation to improve the surgical effect.

In our cases, preoperative MR revealed that the tumor was near the ureter in this case, which was confirmed by intraoperative observation. Therefore, combined with the experience of pelvic surgery in urology, Anorectum and Gynecology,^[19,25,26]^ laparoscopic resection combined with catheter placement under cystoscope was chosen. Before laparoscopy, the double-J catheter was placed in the ureter with a cystoscope. The visual field in the operation field is widened to expose the tumor and its surrounding anatomical structures, the tumor, and surrounding tissues are stripped more safely, and the ureter position is exposed during the operation to avoid intraoperative injury. Moreover, even if there is inevitable lateral injury, the double-J tube can still support and expand the ureter, ensure smooth micturition, and avoid obstruction, hydronephrosis, and renal function damage. In this case, intraoperative blood loss was less, and the patient was discharged 3 days after operation without any special discomfort.

In conclusion, schwannoma of the seminal vesicle is a very rare disease. At present, the diagnosis mainly depends on pathological results. The anatomical position of the seminal vesicle is located in the deep pelvic cavity and adjacent to the ureter. Biopsy and operation are difficult and prone to a side injury. It was found in this case that a double-J tube may effectively expose the relative relationship between the ureter and seminal vesicle and reduce the risk of a side injury. This study is expected to provide doctors with a new treatment option to explore.

## Author contributions

CL, AL designed the study and wrote the manuscript. SL, XW participated in acquisition of clinical data and literatures search. MC, LM supervised research and revised the paper.
